# Defining the gene expression signature of rhabdomyosarcoma by meta-analysis

**DOI:** 10.1186/1471-2164-7-287

**Published:** 2006-11-07

**Authors:** Chiara Romualdi, Cristiano De Pittà, Lucia Tombolan, Stefania Bortoluzzi, Francesca Sartori, Angelo Rosolen, Gerolamo Lanfranchi

**Affiliations:** 1 CRIBI Biotechnology Centre and Biology Department, University of Padova, Padova, Italy; 2 Biology Department, University of Padova, Padova, Italy; 3 Clinica di Oncoematologia Pediatrica, Azienda Ospedaliera-University of Padova, Padova, Italy

## Abstract

**Background:**

Rhabdomyosarcoma is a highly malignant soft tissue sarcoma in childhood and arises as a consequence of regulatory disruption of the growth and differentiation pathways of myogenic precursor cells. The pathogenic pathways involved in this tumor are mostly unknown and therefore a better characterization of RMS gene expression profile would represent a considerable advance. The availability of publicly available gene expression datasets have opened up new challenges especially for the integration of data generated by different research groups and different array platforms with the purpose of obtaining new insights on the biological process investigated.

**Results:**

In this work we performed a meta-analysis on four microarray and two SAGE datasets of gene expression data on RMS in order to evaluate the degree of agreement of the biological results obtained by these different studies and to identify common regulatory pathways that could be responsible of tumor growth. Regulatory pathways and biological processes significantly enriched has been investigated and a list of differentially meta-profiles have been identified as possible candidate of aggressiveness of RMS.

**Conclusion:**

Our results point to a general down regulation of the energy production pathways, suggesting a hypoxic physiology for RMS cells. This result agrees with the high malignancy of RMS and with its resistance to most of the therapeutic treatments. In this context, different isoforms of the *ANT *gene have been consistently identified for the first time as differentially expressed in RMS. This gene is involved in anti-apoptotic processes when cells grow in low oxygen conditions. These new insights in the biological processes responsible of RMS growth and development demonstrate the effective advantage of the use of integrated analysis of gene expression studies.

## Background

Rhabdomyosarcoma (RMS) is the most common soft tissue sarcoma in childhood while is rare but highly malignant in adults. RMS arises as a consequence of regulatory disruption of the growth and differentiation pathways of myogenic precursor cells. Although in the last few years several functional and genomic studies have been published on RMS, many issues are still to be clarified such as the identity of the myogenic precursor cell of RMS [1].

Recently, many studies focused on cancer have demonstrated the great potential of the genomic approach based on DNA microarray and chips for the identification of new regulatory pathways involved in complex biological processes like tumor development, proliferation [2] and metastasis [3].

Among the genomic technologies for the study of cell transcriptome at global level, DNA microarray and serial analysis of gene expression (SAGE) methodologies have already been used to study gene alterations in RMS. Khan and colleagues [4] applied cDNA microarray technology to find new potential markers able to distinguish different types of small round blue cell tumors (SRBCT) including 26 samples of RMS. More recently, Baer research group [5] investigated, with Affymetrix chips, the expression signatures of 12 primary pediatric RMS and 11 Ewing's sarcomas.

Rhabdomyosarcomas are classified in two main subtypes: embryonal RMS (ERMS) and alveolar RMS (ARMS). ARMS represents approximately 25–30% of RMS and has a worse prognosis than ERMS. Cytogenetic and molecular analyses have demonstrated that ARMS frequently harbors two reciprocal chromosomal translocations t(2;13)(q35;q14) or t(1;13)(p36;q14), in which *PAX3 *or *PAX7 *and *FKHR *genes are juxtaposed producing two novel fusion transcription factors PAX3-FKHR or PAX7-FKHR. In relation to this classification, two genomic studies have been recently published. Wachtel and co-workers [6] studied the similarities in the gene expression profiles among 29 different RMSs, including ERMSs, translocation negative and positive ARMSs using a genome-wide Affymetrix chip. De Pittà et al. [7] adopted a custom muscle-specific cDNA platform to compare the gene expression signatures of translocation positive and negative RMS samples for the identification of possible marker genes. Finally, with the SAGE technique, Schaaf et al. [8] identified genes differentially expressed in ERMS and/or ARMS as compared with three different reference samples: old, young and fetal skeletal muscle.

During the reviewing process of this manuscript a new study on RMS expression profiles has been published [9]. Davicioni and Collaborators try to test what extent of PAX-FKHR determine class and behaviors of ARMS using oligonucleotide microarrays expression profiling of 139 primary RMS tumors and an *in vitro *model. We decided to discuss and include this work in the paper as a further evaluation of our results.

The availability of public gene expression repositories has opened up a new realm of possibilities especially for microarray data analysis. An essential challenge, in fact, is given by the efficient integration of gene expression data on the same issue generated by different research groups using different array platforms and experimental conditions. The data integration is done in order to confirm and strengthen the results of single studies as well as to find or complete common cellular pathways that are found altered in specific physiological or pathological processes under study. Pivotal studies of this type have been performed on cancer [10]. The analysis of multiple gene expression datasets concerning a common biological problem, called "*meta-analysis*", has shown the capability of retrieving much more relevant information than single experiment datasets [11-13].

Here we present the results of a comprehensive meta-analysis that has been performed on all the available gene expression datasets published on RMS in order: *i*) to evaluate the degree of agreement of the biological results obtained by these different studies and, *ii*) to identify common regulatory pathways that could be relevant for the pathophysiology of RMS. In this study we have adopted a robust statistical approach, similar to that proposed by Rhodes and colleagues [10], for the analysis of four DNA microarray/chip and two SAGE datasets published on RMS. Two different approaches have been used for our meta-analysis: the "single dataset" and the "matching" approach. In the former approach, each dataset is analyzed individually and Gene Ontology categories (GO) and/or metabolic pathways (obtained by KEGG databases) commonly enriched in most of the studies are further investigated.

The low data reproducibility among microarray experiments performed with different platforms and different statistical analysis has recently stressed the strong variability that seems intrinsic to this technology. In spite of this variation, the classification of genes for which probes are present in different platforms into functional categories or pathways has demonstrated to favor the comparison of the expression data obtained in different studies, despite substantial differences in the lists of differentially expressed genes identified [14].

In our comparative analysis of RMS expression datasets we have adopted such gene "matching" approach, selecting a set of "core" genes common to all platforms, matched by Entrez Gene, and performing statistical analyses on this gene core. Our results indicate a general down regulation of pathways involved in energy and carbohydrate metabolism. In particular, oxidative phosphorylation, ATP synthesis, Krebs cycle pathways and several genes with biological and molecular functions related to ATP production and oxidoreductase activity show decreased level of expression with respect to healthy skeletal muscle tissues. Furthermore, the list of differentially expressed genes in common across the studies seems to confirm and complete this biological aspect of the disease. In particular, the underexpression of genes for energy production can be only partly due to the presence of undifferentiated muscle tissues in RMS patients. Our results, in fact, seem to suggest an important role for the hypoxic cellular condition in the anti-apoptotic process and then in the proliferation and growth of the tumor. This could justify the highly aggressive and malignant phenotype typical of solid tumors like RMS.

Supplementary information for the results obtained by the meta-analysis can be browsed at [15].

## Results and discussion

RMS is of great interest as a cancer model, not only for its mostly unknown pathogenic pathway, but also for its implication in cellular differentiation processes. Therefore, a better characterization of the biological and molecular processes and of the networks of interactions involved in regulation of RMS gene expression would represent a considerable advance in basic and translational research regarding this tumor. In this context, integrative bioinformatics analysis using different sources of information, has demonstrated to be a promising approach for a better comprehension of pathogenic processes otherwise not apparent from standard analysis methods. Technical differences between microarray platforms and biological variability of samples can lead to differences and fluctuation in gene expression profiles. *Meta-analysis *on expression data can give a more robust and complete insight on the biological process investigated, overcoming these problems. In this work we performed a meta-analysis on gene expression datasets obtained by four projects that applied DNA microarray/chip technology and two projects that used SAGE technology to study RMS (Table 1).

### Meta-analysis on RMS expression data

Two different approaches have been used for data analysis. The "Single dataset" approach performs statistical test individually in each study and focuses the scientific investigation on functional categories of deregulated genes that are common to most of the studies. The "Matching" approach uses Entrez Gene ID for the generation of a matched dataset where *meta-profiles *are used for the identification of common deregulated genes.

#### Single dataset approach

**Figure 1:**
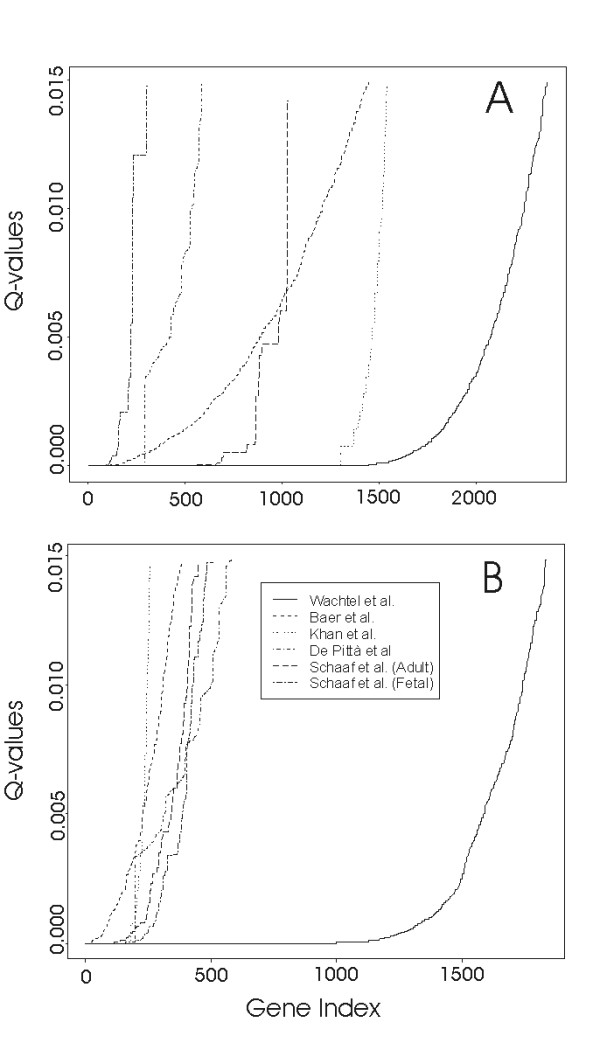
False discovery rate (FDR) trend in each dataset. On the x-axis genes are ranked according to the p-values obtained by statistical test, while on the y-axis the Q-value (FDR) is reported. Panel A: underexpressed genes; panel B: overexpressed genes.

The results of each individual expression study have been evaluated under the testing assumption of no relationship between expression values of a gene and the distinction between RMS and healthy tissue. *P-values *resulting from statistical tests have been assigned to all genes and then, after sorting them through *p-values*, *q*-*values *(false discovery rate, FDR) [16] were calculated and associated to genes. The FDR threshold for the identification of differentially expressed genes has been set at 0.01. Figure 1 shows the *q*-*values *plots of the six datasets analyzed. The X-axis is the rank index for genes sorted by *p*-*values*. Given a specific value of *q*, the intersection with the curves shows the number of genes identified as differentially under- (panel A) or overexpressed (panel B) in each dataset. Table 2 lists the total numbers and percentages of differentially expressed genes resulting from the meta-analysis of each single RMS dataset. From Fig. 1A,B and Table 2 it is evident that RMS results in a general down regulation of the transcriptome: 4 out of 5 studies (Khan, Baer, Wachtel, Schaaf datasets) detect a higher number of underexpressed genes and the percentage they represent in the different platforms is comparable with the exception of Khan dataset, which results in a higher difference (23% underexpressed and 4% overexpressed). This discrepancy could results from differences in the platforms. As suggested by Rhodes et al. [11] the number of differentially expressed genes at a given *q *value is highly influenced by the total number of probes and samples as well as by more general issues like the nature of probe sequences, array quality, etc.

Differentially expressed genes have been grouped into functional categories and pathways according to GO and KEGG, respectively. Then, GO and KEGG classes that show altered genes in a number that is significantly enriched in comparison to a random distribution have been calculated for each platform. Significant classes with FDR less than 0.05 have been compared across the studies and ranked according to the number studies that appear to share them. In the present approach, GO categories and KEGG pathways represent statistical and biological units. Focusing on these macro structures (partially overlapped because the same gene product can be associated to various biological functions and can be involved in more than one metabolic pathway), it is possible to integrate results coming from different studies achieving a comprehensive picture of the biological processes involved The picture could emerge only partially using each dataset individually. Differences among different expression signatures can be generated by insufficient resolution power of the statistical analysis applied, but also by the use of different reference samples (see Methods for details). It should be noted in fact that skeletal muscle is a composite tissue with a dominant component of myofibres and satellite cells plus connective and adipose tissues, blood cells etc. Therefore, the contribution of different "contaminant" cells to reference RNA preparations could be slightly different between normal muscle controls. However, we believe that the key deregulated pathways responsible of the muscle pathology should be evident in most of the studies whatever the healthy muscle reference. In this context meta-analysis is helpful to discover and highlight the core biological aspects of the pathology.

Table 3 summarizes the result of this process: the GO and KEGG gene categories that show an enriched presence in at least four independent datasets are here indicated. In this representation, different colors represent the expression levels of genes belonging to each category (green: under-expression, red: over expression, black: mixture of under and over expression). As a general overview, we can conclude that most of the underexpressed genes codify for products involved in energy production (ATP synthesis, oxidoreductase activity, electron transport, oxidative phosphorylation) and energy/carbohydrate metabolism (ATP synthesis, oxidative phosphorylation, Krebs cycle, carbon fixation).

The list of differentially expressed genes and of the enriched GO and KEGG classes in the study of Davicioni et al. [9] has been reported in Additional file 1. This new analysis seems to validate our findings: all the molecular and biological functions and the metabolic pathways cited above are confirmed as significantly enriched and downregulated.

**Figure 2:**
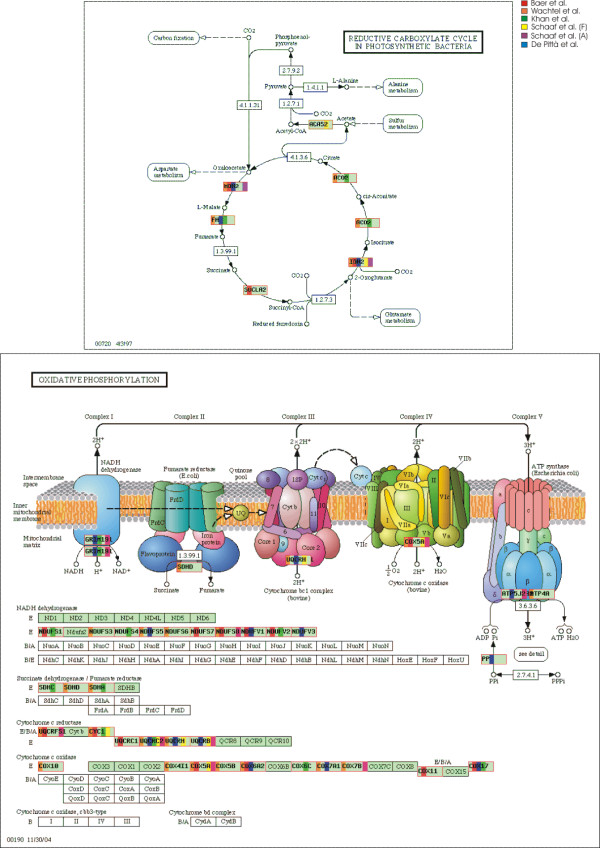
Integrated metabolic map (from KEGG database) of Kreb cycle and Oxidative phosphorialtion. Boxes in red represent gene products that are differentially underexpressed in at least one dataset. Colored bands inside red boxes correspond to the dataset in which the product gene is differentially expressed (according to the legend).

To further investigate each pathway, we have generated integrated KEGG maps with differentially expressed genes identified in the different studies. Figure 2, for instance, shows the integrated map of oxidative phosphorylation and Krebs cycle, as obtained by the meta-analysis (data from the six studies are shown in different colors). This example clearly illustrates the results that are achieved with our meta-analysis. In that particular functional maps in fact, each single dataset individually examined gives only a partial picture of these deregulated metabolic pathways. The integration of all datasets fills in the picture with gene members that are not revealed by single analysis, providing a comprehensive evaluation of the deregulation of that metabolic process.

RMS is characterized by the presence of undifferentiated muscle tissues, and therefore ATP production appears to be decreased with respect to normal skeletal muscle tissue. In this context, the metabolic pathways described before that appears strongly deregulated from our analysis can be considered as a consequence of the presence of such undifferentiated cells. Nevertheless, absence of oxygen has been recently associated to tumor progression and growth. It is well known that cells undergo a variety of biological responses when placed in hypoxic conditions, including activation of signaling pathways that regulate proliferation, angiogenesis and death. Cancer cells may have an adaptive behavior allowing tumors to survive and grow under hypoxia, leading to worse prognosis and resistance to radiation therapy [17] as observed in RMS. The hypoxic phenotype has also been found in neuroblastoma [18], an early childhood tumor that shares several features with RMS. Axelson et al. [18] suggested that hypoxia condition contributes to cell de-differentiation and tumor heterogeneity, even though the molecular mechanism underlying this process is still undefined. Distinct tumor cells show a different response in hypoxic condition. This last phenomenon is particularly marked in solid tumors and specifically in RMS. Then, the results obtained with our meta-analysis about energy production down regulation can be thought as a consequence of the pathology progress but also as a cause of the heterogeneity and aggressiveness of the tumor.

The complete set of integrated KEGG maps significantly enriched with genes altered in RMS is available in the Supplementary Information.

#### Matching approach

We performed statistical analysis on sets of gene probes common to all platforms examined in our study, matched according to Entrez Gene (EG) entries. Table 4 shows the distribution of EG entries in each platform (total number of EG entries, number of singleton EG and number of probes not matching any EG entry) and the redundancy measure (RD) value. Furthermore, EG entries common to 2, 3, 4 and 5 datasets respectively, are shown in Table 5. Additional file 2 reports the pairwise number of overlapping EG entries in the five platforms.

**Figure 3:**
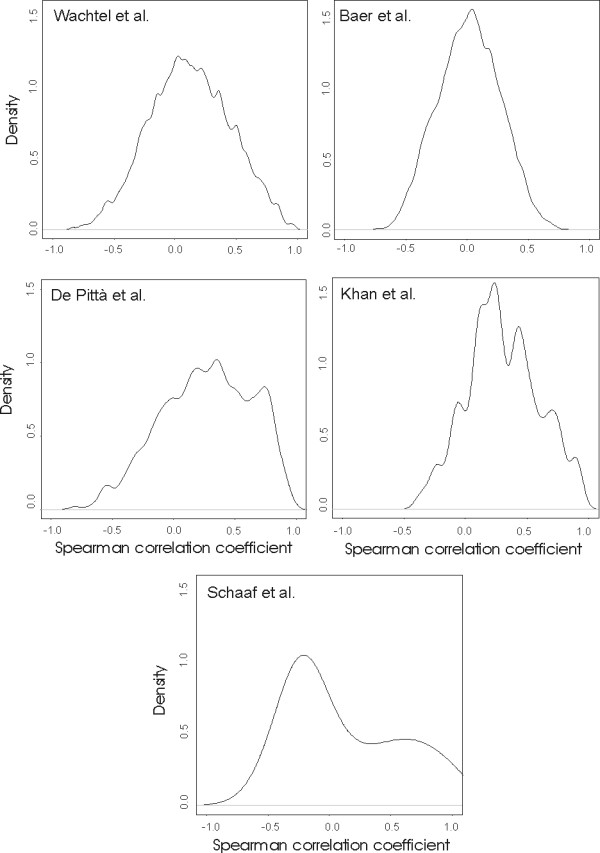
Frequency distribution of the mean pairwise Spearman correlation coefficient obtained from the comparison (with a jackknives procedure) of the expression profiles of probes belonging to the same Entrez Gene.

All the expression datasets derived from microarray platforms show a similar level of RD (see the Methods for details) while, as expected, the SAGE dataset (composed by some 46,000 tags) has a significantly higher RD value. On the other hand, Figure 3 shows the distribution of the platform reproducibility index (RP), defined as the mean correlation of pairwise expression profiles of probes assigned to the same EG entry for each platform (see the Methods for details). We found that cDNA platforms show a distribution centered on RP value of 0.3–0.4, while Affymetrix platforms of 0.0–0.1. Probes in the Affimetrix platform are synthesized as pairs of short oligonucleotides: each couple consists of an oligonucleotide perfectly matched to the target sequence and a twin oligonucleotide mismatched at a single position. This probe configuration should give a higher specificity of probes for the identification of transcript variants and alternative splicing isoforms. On the other hand, cDNA platforms contain probes with long sequences and, even if designed on the 3' or 5' portions of transcripts, could result in less specific hybridizations with targets. Our results are in agreement with those obtained by Lee et al. where UniGene clusters were used to match the probes of platforms [19]. The variability in the expression profiles of genes belonging to the same EG entry introduces a degree of complexity in the comparison of probes across different array platforms. In this context, the introduction of *meta-profiles *(see the Methods section) allows us the comparison of EG entries across different studies. Then, a *meta-profile *is defined as the trimmed median profile of the probes annotated with the same EG and is virtually representative of that gene. After identifying EG entries common to all the six studies, statistical tests (permutational t-test for microarray data and Audic and Claverie test for SAGE data) have been performed on *meta-profiles*.

### Meta-profiles of differentially expressed genes

**Figure 4:**
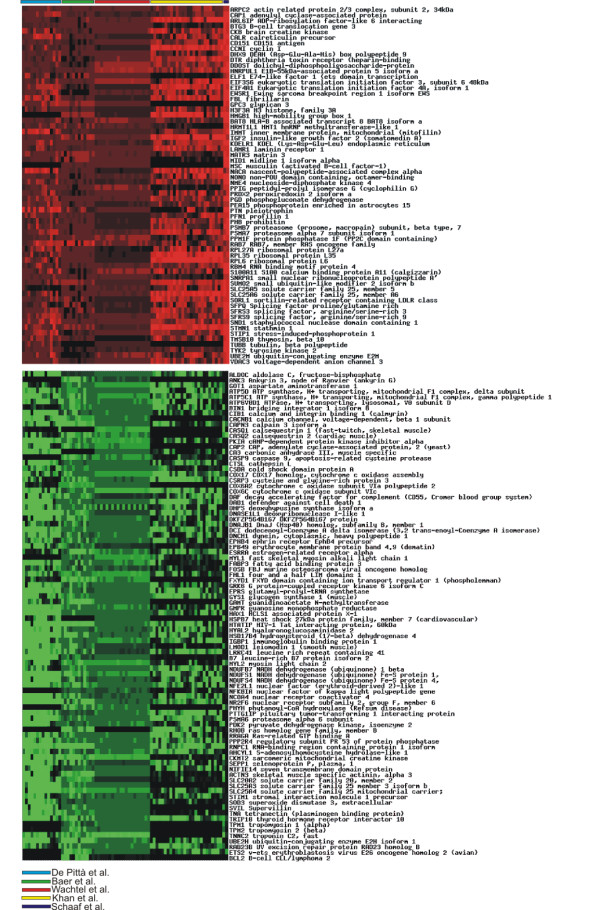
Genes differentially expressed in at least five datasets using meta-profiles.

Figure 4 shows the *meta-profiles *of the genes that were established as under- (88) and overexpressed (65) in at least five RMS expression datasets. Several of these genes have already been found deregulated in RMS and are mostly defined as anti-apoptosis or are involved in tumor progression and growth as well as in muscle differentiation and sarcomere morphogenesis [see Additional file 3 for the complete list of the 153 differentially expressed genes].

Of these 153 genes on 5,500 identified as differentially expressed (FDR< 0.1), 52 (34%, p = 0.003, 41 down and 11 up regulated) are confirmed in Davicioni et al. work if considering all the 121 RMS tumors. On the other hand, taking into account only genes differentially expressed on the 102 ARMS and ERMS Davicioni's samples (eliminating all the other RMS subtypes) the number of confirmed genes increases to 73 (48%, p = 0.01, 55 down and 23 up regulated) [Additional files 1 and 3]. The statistical significance reported above was calculated considering the probability of selecting by chance in the list of differentially expressed genes exactly 52 and 73 genes in common with the 153 previously identified (hypergeometric distribution).

In particular we would like to discuss some of the most interesting of these deregulated genes.

*IGF2 *seems to be an important regulator of cell growth, differentiation, and survival through endocrine and paracrine/autocrine signaling [20,21]. The widely accepted hypothesis is that *IGF2 *is maternally imprinted and that disomy at 11p15 may lead to increased expression of *IGF2 *from two active paternal alleles [22]. It is interesting to note that *IGF2 *is validated by the study of Davicioni et al. only taking into account ARMS and ERMS samples. *STIM1 *(stromal interaction molecule) is a candidate tumor suppressor gene mapping to human chromosome 11p15.5, a region that has been associated to a variety of cancers, including the ERMS. The transfection of *STIM1 *cDNA into either rhabdoid tumor or RMS cell lines, where the endogenous transcript is not detectable, results in the inhibition of cell growth and the stimulation of cell death [23]. In rodent myoblast cell lines, the expression of cloned *STIMI *gene causes withdrawal from cell cycle independent from differentiation. These data suggest *STIM1 *as a candidate tumor suppressor gene. Our results are consistent with the hypothesis that this gene has a crucial role in the pathogenesis of rhabdomyosarcoma. *STIM1 *is validated by Davicioni's work only for ARMS and ERMS samples. The skeletal muscle Lim protein 1 (SLIM1, alias FHL1) is highly expressed in skeletal muscle [24] and contains four- and- a- half LIM domains that play a critical role in tissue differentiation and cytoskeletal integrity [25]. In the model myogenic cell line C2C12, in fact, it has been demonstrated that the overexpression of FHL1 protein promotes differentiation [26]. Furthermore, a recent study suggests a role for FHL1 in sarcomere assembly [27]. Davicioni et al. data confirm *FHL1 *as significantly downregulated using both ARMS and ERMS samples or the complete set of samples.

The products of most of the underexpressed genes in RMS resulting from meta-analysis, are localized in the mitochondrion [see Additional file 3] and are mainly involved in aerobic energy metabolism. These results seem to complete and better delineate the disease picture obtained with the previous approach ("single dataset") especially with regards to metabolic pathways of cell response to hypoxia. Among these genes, the mitochondrial adenine nucleotide translocator (ANT) appears to act as a bi-functional protein catalyzing ADP/ATP exchanges across the mitochondrial inner membrane and playing an essential role in aerobic energy metabolism from one side, and from another side, being converted into pro-apoptotic pore under the control of onco- and anti-oncoproteins of the Bax/Bcl2 family [28-30]. In humans there are three closely related isoforms of ANT and their expression depends on cell type, differentiation stage, and metabolic state. *SCL25A4 *(alias *ANT1*) is mainly expressed in the heart, skeletal muscle and to a lesser extent in brain [31]. *SCL25A5 *(alias *ANT2*) is growth-dependent and marker of cell proliferation [32], while *SCL25A6 *(alias *ANT3*) is ubiquitously expressed. The induction of *ANT2 *expression in cancer cells is directly related to their glycolytic metabolism that is activated when occurs an interruption of aerobic energy metabolism or an oxidative stress [33]. ANT2 has been suggested to act in a reverse manner importing ATP into mitochondria and exporting ADP in order to preserve the mitochondrial Δψ preventing apoptosis [34]. Previous studies have shown that over-expression of ANT1 and ANT3 in some cell lines produce a rapid cell death, with a concomitant decrease in mitochondrial Δψ and an increase in nucleosomal DNA degradation [35], while in other cell types the over-expression of these isoforms do not induce apoptosis. This may suggest that ANT isoforms can induce apoptosis when expressed in the wrong place or at the wrong time. In a recent work, Chevrollier and colleagues [34] demonstrated that the *ANT2 *and *ANT3 *genes are co-expressed in tumor cells as well as in non transformed cells. Our indication of a general under expression of aerobic energy metabolism is in agreement with the functions and the behavior of ANT isoforms. In fact, *ANT2 *and *ANT3 *are found to be both overexpressed while *ANT1*, the skeletal muscle specific isoform, underexpressed. Then, our results, are in agreement with the hypothesis of the bi-functional role of ANT isoforms that promote glycolytic metabolism and at the same time prevent apoptosis increasing cell proliferation. The differential expression of *ANT1*, *ANT2 *and *ANT3 *is confirmed by the work of Davicioni and Collaborators.

Very recently it has been shown that low oxygen tension in tumors was associated with increased metastasis and poor survival in patients suffering from head, neck, cervical or breast cancer [36]. Hypoxia-inducible factor-1-α (HIF-1α) is the key transcription factor that is induced by hypoxia and regulated by a proline hydroxylase. HIF-1 activates the transcription of a series of genes that are involved in crucial aspects of cancer biology including angiogenesis, cell survival, glucose metabolism and invasion. Although our results seem to suggest a general hypoxic state in RMS cells, neither *HIF-1 *nor other genes directly regulated by HIF-1 have been found consistently deregulated. There are no evidences for the interactions between HIF-1 and *ANT *gene, and therefore what we see in RMS could only be related to a specific pathway of the cellular response in case of low level of oxygen: the process of anti-apoptosis.

Other differentially expressed genes appear to be involved in cell growth and/or maintenance, and therefore could play an important role in RMS biology. In this context two interesting genes, involved in cell proliferation and apoptosis, *CD151 *antigen (*CD151*) and ADP-ribosylation factor-like 6 interacting protein (*ARL6IP *alias *ARMER*) were found overexpressed. *CD151*, identified as differentially expressed also in Davicioni's data, mediates signal transduction events that play a role in the regulation of cell development, activation, growth and motility [37]. CD151 is the first member of the tetraspanin family that has been suggested as a promoter of human tumor metastasis [38]. For a number of malignant diseases the level of expression of members of the tetraspanin family was found to correlate with tumor cell invasiveness, ability to form metastases, and poor clinical outcome [39,40]. ARL6IP (alias ARMER) is an apoptotic regulator located in the membrane of the endoplasmic reticulum [41] that protects cells by inhibiting caspase-9 activity. In fact cells in which *ARMER *was overexpressed exhibited protection from multiple apoptotic inducers including serum starvation, UV irradiation and tumor necrosis factor alpha [42].

According to Segal et al. [2] work on conditional activity of expression modules in cancer, liver, CNS and prostate tumors share regulatory modules implicated in energy metabolism. This may suggest the possibility of common biological processes among these solid tumors and RMS specifically involved in tumor growth and aggressiveness.

## Conclusion

In this work we have identified common altered biological pathways consistent with a hypoxic physiology of RMS. Hypoxic physiology has recently been associated to poor patient outcome in several types of tumors [36]. This result is coherent with the high malignancy of RMS and with its resistance to several kinds of therapeutic treatments, however none of the expression studies used in our meta-analysis, when analyzed separately, has the power to capture this aspect of the pathology. Furthermore, new genes involved in the anti-apoptotic process like the *ANT *family genes have been identified for the first time in RMS by this meta-analysis. Different isoforms of *ANT *gene, in fact, seem to play an important role in the process of cellular proliferation and tumor growth.

A further support to our findings has been obtained by analyzing the new study of Davicioni and colleagues published during the reviewing process of our manuscript. Most of the *meta-profiles *found differentially expressed and all the enriched Gene Ontology and KEGG classes discussed here appear to be confirmed by this new work.

Our results have also confirmed the great potential of a combined and integrated statistical/bioinformatic analysis of expression datasets for the investigation of complex transcriptomes, such as those of highly malignant tumors. In the last few years we have witnessed discordant opinions with regards to microarray data. Intrinsic technological differences and biological variability will always lead to the problem of results variability and low reproducibility. Nevertheless, the availability of gene expression data compliant to the common standard guidelines as those proposed by MGED society [43], and the possibility to perform comparative/integrative analysis will help researchers to have a more comprehensive and robust insight into the biological problem investigated.

## Methods

### Data collection

Data used in this study are either publicly available or have been generously provided by the Authors. Wachtel et al. [6] dataset is based on Affymetrix chips (HG-U133A) and is available at EBI ArrayExpress database (identification number: E-MEXP-121). This dataset consists of 29 experiments, 14 expression profiles of alveolar rhabdomyosarcoma (ARMS) and 15 of embrional rhabdomyosarcoma (ERMS). The dataset produced by Baer et al. [5] is based on Affymetrix technology (U95Av2) and is available on GEO database (GSE967 series identification number). The Authors produced expression profiles of 12 RMS samples. Khan et al [4] dataset has been obtained on custom cDNA microarray platform of 6,567 probes [44] and consists of 26 RMS samples from cell lines and biopsies. De Pittà et al. [7] used a custom cDNA muscle array ([45], GEO database, GPL2011 platforms and GSE2787 series identification number) of 4,992 transcripts and compared expression profiles of 14 ARMS (7 translocation positive and 7 negative). Finally, Schaaf et al. [8] dataset, kindly provided by the authors, includes 3 RMS samples (2 ARMS and 1 ERMS) and 3 healthy skeletal muscle samples (fetal, young and old muscles). The SAGE libraries considered in our study consist of 46,445 tags. To take advantage of these multiple references we have considered the results of Schaaf et al. data as two distinct datasets: one with fetal and one with adult (young and old) skeletal muscle profiles as control reference called respectively "Schaaf et al. Fetal" and "Schaaf et al. Adult". In this way we have obtained two lists of differentially expressed genes that are reported in Table 2 as Schaaf et al. (Fetal) and Schaaf et al. (Adult) obtained by the comparison of all the three libraries of RMS vs fetal and vs adult.

Davicioni et al. [9] used Affymetrix chips (HG-U133A) on 121 RMS primary tumor samples (102 ARMS and ERMS plus 19 other RMS subtypes) and 30 tumor cell lines and their data are available at the National Cancer Institute Cancer Array Database [46].

Expression datasets obtained with Affymerix chips have been normalized with the invariant method [47] using dChip software [48]. After normalization, 6 RMS microarray experiments (RMS01, RMS02, RMS03, RMS04, RMS05, RMS09) in Wachtel et al. dataset and 12 in Davicioni et al. dataset (A377, A343, A355, A403, A412, A423, A425, A467, A860, B638, B648, B655) have been eliminated from the analysis because not of the best quality. cDNA microarrays datasets were normalized firstly with with global and then with LOWESS statistical procedures [49] using MIDAW web tool [50,51], while SAGE libraries have been normalized through the total number of tag *per *library.

Affymetrix platforms measure the absolute expression for each probe, while cDNA microarrays provide a relative measure of each transcript in two compared conditions. To compare the expression values of all the studies, we have used as common reference four gene expression dataset produced with healthy skeletal muscle. They are deposited in GEO database with the following accession numbers: GSM19013 and GSM19014 produced with HG-U133A Affimetrix chip platform [52], GSM12674 and GSM12693 produced with U95Av2 platform [53].

On the other hand, Khan et al. study used in the competitive hybridization mRNA from RMS biopsy and mRNA from a tissue pool of skin and muscle from a 13-week male fetus, while De Pittà et al. used as common reference a commercial RNA (Stratagene, Europe) prepared from male fetal skeletal muscle.

### Meta-analysis

As proposed by Rhodes et al. [10], for each individual study log2 transformation of gene expression ratio has been considered and a permutational t-test has been performed. P-values and Q-values (false discovery rate, FDR) [16] have been used as ranking. Q-values for each gene has been defined as: Q = (p*n)/*i*, where *p *is the *p*-value of the gene, *n *the total number of genes and *i *is the number of genes at or better than *p*.

For SAGE data (tag counts) specific statistical tests have been proposed for the identification of differentially expressed genes. Audic and Claverie test and the chi-squared test [54] have been performed with IDEG6 web tool [55,56].

In the individual analysis of each single dataset FDR threshold for the identification of differentially expressed genes has been set to 0.01.

On the other hand, when considering matched dataset according to Entrez Gene (see Probe Matching paragraph for matching details) a summary statistic, called S, (Fisher's inverse chi-squared method, [57]) was computed using the *p*-values of the statistical tests of that gene in each study:

S = - 2 * log(*p*_1_) - 2 * log(*p*_2_) - 2 * log(*p*_3_) - ... - 2 * log(*p*_*n*_),

where *n *is the total number of studies.

As proposed by Moreau et al. [58] we used a parametric approach that compares the statistic S with a chi-square distribution with *2n *degrees of freedom. Then, genes where ranked according to S. This procedure has been applied not only on those genes common to all studies, but also separately on genes common to 2, 3, 4 and 5 studies (but absent in the others).

All the statistical analyses have been performed with custom Perl functions and R/Bioconductor packages [59].

### Annotation

Differentially expressed gene has been associated to one or more Gene Ontology (GO) categories and KEGG metabolic pathways [60] using DAVID tool [61,62]. Class enrichment (with respect to the entire platform) has been calculated with the hypergeometric distribution (Fisher exact test) [63]. The hypergeometric distribution is used to obtain the chance probability of observing the number of genes from a particular GO/KEGG category among the selected differentially expressed genes. The probability P of observing at least *k *genes of a functional category within a group of *n *genes is given by:

P=∑i=kn(fi)(g−fn−i)(gn)
 MathType@MTEF@5@5@+=feaafiart1ev1aaatCvAUfKttLearuWrP9MDH5MBPbIqV92AaeXatLxBI9gBaebbnrfifHhDYfgasaacH8akY=wiFfYdH8Gipec8Eeeu0xXdbba9frFj0=OqFfea0dXdd9vqai=hGuQ8kuc9pgc9s8qqaq=dirpe0xb9q8qiLsFr0=vr0=vr0dc8meaabaqaciaacaGaaeqabaqabeGadaaakeaacqWGqbaucqGH9aqpdaaeWbqaamaalaaabaWaaeWaaeaafaqabeGabaaabaGaemOzaygabaGaemyAaKgaaaGaayjkaiaawMcaamaabmaabaqbaeqabiqaaaqaaiabdEgaNjabgkHiTiabdAgaMbqaaiabd6gaUjabgkHiTiabdMgaPbaaaiaawIcacaGLPaaaaeaadaqadaqaauaabeqaceaaaeaacqWGNbWzaeaacqWGUbGBaaaacaGLOaGaayzkaaaaaaWcbaGaemyAaKMaeyypa0Jaem4AaSgabaGaemOBa4ganiabggHiLdaaaa@47C6@

where *f *is the total number of genes with the same GO class (in the microarray platform) and *g *is the total number of genes within our platform.

Then, the lists of the significantly enriched GO categories and KEGG pathways (FDR = 0.05) for each study have been compared, and only those common to at least 4 studies has been considered as relevant for RMS.

### Metabolic map

Dedicated Perl and PHP scripts have been implemented (i) to identify on KEGG maps differentially expressed genes product coordinates and (ii) to paint gene products boxes with different colors representing the five different studies.

### Probe matching

The identification of the common probe set shared by the platforms used in the different studies has been obtained through the Entrez Gene accession number.

Platform redundancy (RD) is defined as the mean number (after the elimination of singleton Entrez Gene) of multiple probes representative of the same gene. RD measures for each platform are reported in Table 4.

On the other hand, platform reproducibility (RP) is defined as the mean Spearman correlation among expression profiles of probes representative of the same gene. Probes that identify the same gene can have different expression profiles. This phenomenon, given either by the presence of alternative splicings and by the intrinsic errors associated to each measures, can be tricky when comparing expression values across different studies.

To work out this problem, we have generated for each Entrez Gene a "*meta-profile*" obtained as described in the following. Given a specific Entrez Gene, the average of all the pair-wise Spearman correlations between the expression profile of one probe in this Entrez Gene class and the median profile of the corresponding ID without the first gene (jackknife procedure) was calculated. Those probes with a Spearman correlation less than 0.5 are eliminated and the median expression profile recalculated. At the end of the procedure, a *meta-profile *is the median profile of the remaining probes. Then for each Entrez Gene we have a trimmed expression profile – called *meta-profile *– that is virtually representative of that gene. Then, *meta-profiles *that generate matched dataset have been used for the comparison of the expression profiles among studies using the "matching" approach.

## Authors' contributions

CR conceived the study, performed all the statistical and bioinformatic analysis (normalization, statistical test, metabolic pathways and biological processes enrichment) and drafted the manuscript. CDP, LT, SB participated in the design of the study, revised the manuscript and participated in the investigation of the differentially expressed genes and interpretation of the results. FS participated in the revision of the manuscript. AR and GL supervised the study, partecipating the design and coordination of the work, the interpretation of the results and revision of the manuscript. All authors read and approved the final version of the manuscript.

## Supplementary Material

Additional File 1 Complete list of differentially expressed genes and GO/KEGG enriched classes found in Davicioni et al. (2006) work. Two lists of differentially expressed genes are reported: the first one includes genes up- and down-regulated in all the 121 RMS samples and the second one includes deregulated genes only in the 102 ARMS and ERMS samples. GO and KEGG enriched classes are reported only for the second list. Click here for file

Additional File 2 Percentage and actual number of Entrez Genes entries shared by the studies included in the meta-analysis. Percentages are calculated according to the total number of probes *per *row. Click here for file

Additional File 3 Complete list of differentially expressed *meta-profiles*. D1 and D2 columns with gray cells represents genes found differentially expressed in Davicioni et al. (2006) study. D1 column shows genes differentially expressed taking into account only embrional and alveolar RMS, while D2 shows gene differentially expressed with all the samples in Davicioni's paper. Click here for file

## Figures and Tables

**Table 1:** Rhabdomyosarcoma gene expression datasets used in the meta-analysis

**Authors**	**Journal**	**Year**	**Array platform**	**Total N. probes**	**N. of RMS samples**
Wachtel *et al*.	*Cancer Research*	2004	Oligo Affymetrix	22,283	29
Baer *et al*.	*Int. J. Cancer*	2004	Oligo Affymetrix	12,558	12
De Pittà *et al*.	*Int. J. Cancer*	2006	cDNA	4,992	14
Khan *et al*.	*Nature Medicine*	2001	cDNA	6,567	26
Schaaf *et al*.	*FASEB J*.	2005	SAGE	46,445	3

**Table 2:** Number and percentage of differentially expressed genes (FDR = 0.01) in each dataset.

**Authors**	**Overexpressed genes**		**Underexpressed genes**	
	*Actual number*	*Percentage*	*Actual number*	*Percentage*
Wachtel *et al*.	1,837	8.3	2,374	10.7
Baer *et al*.	384	3.0	1,453	11.5
De Pittà *et al*.	585	11.7	593	11.8
Khan *et al*.	259	4.0	1,542	23.4
Schaaf *et al*. (Adult)	205	3.0	698	11.3
Schaaf *et al*. (Fetal)	344	5.6	531	8.6

**Table 3:** Gene Ontology categories and KEGG metabolic pathways that have been found significantly enriched in more than 2 expression studies on RMS by the meta-analysis.

**Gene Ontology**	**Class description**^§^	**W**^**$**^	**B**	**DP**	**K**	**SA**	**SF**	**# studies**
**Biological Process**	Cell killing		**x**	**x**	**x**	**x**	**x**	5
	Muscle contraction	**x**	**x**	**x**		**x**	**x**	5
	Biosynthesis	**x**			**x**	**x**	**x**	4
	Appendage morphogenesis	**x**		**x**		**x**	**x**	4
	Macromolecule metabolism	**x**	**x**			**x**	**x**	4
	Myoblast differentiation		**x**		**x**	**x**	**x**	4
	Endothelial cell differentiation	**x**			**x**	**x**	**x**	4
	Regulation of organismal physiological process		**x**	**x**		**x**	**x**	4

**Molecular Functions**	↓ Oxidoreductase activity, acting on the CH-NH group of donors	**x**	**x**	**x**	**x**	**x**	**x**	6
	↓ AMP deaminase activity		**x**	**x**	**x**	**x**	**x**	5
	Thioredoxin-disulfide reductase activity	**x**	**x**		**x**	**x**	**x**	5
	Cytoskeletal protein binding	**x**	**x**	**x**		**x**	**x**	5
	Primary active transporter activity	**x**	**x**	**x**		**x**	**x**	5
	↑ Phospholipid-hydroperoxide glutathione peroxidase activity	**x**	**x**		**x**	**x**	**x**	5
	RNA binding	**x**		**x**		**x**	**x**	4
	4-alpha-glucanotransferase activity		**x**		**x**	**x**	**x**	4
	Amylo-alpha-1,6-glucosidase activity		**x**		**x**	**x**	**x**	4
	↑ Glutathione peroxidase activity		**x**		**x**	**x**	**x**	4
	Protein C-terminus binding		**x**	**x**		**x**	**x**	4
	Ornithine decarboxylase inhibitor activity		**x**		**x**	**x**	**x**	4
	Heme-copper terminal oxidase activity	**x**	**x**			**x**	**x**	4
	↓ Oxidoreductase activity, acting on NADH or NADPH	**x**	**x**			**x**	**x**	4
	↓ Oxidoreductase activity, acting on sulfur group of donors		**x**		**x**	**x**	**x**	4
	↓ Oxidoreductase activity, acting on heme group of donors	**x**	**x**			**x**	**x**	4
	Hydrolase activity, acting on ether bonds		**x**	**x**		**x**	**x**	4
	Intramolecular oxidoreductase activity	**x**	**x**			**x**	**x**	4
	↑ Phosphatase activator activity		**x**	**x**		**x**	**x**	4
	↓ Electron transporter, transferring electrons from coqh2-cytochrome c reductase complex and cytochrome c oxidase complex activity	**x**	**x**		**x**		**x**	4

**Cellular Component**	↓ Proton-transporting ATP synthase complex (sensu Eukaryota)	**x**		**x**	**x**	**x**		4
	↓ Proton-transporting ATP synthase complex, catalytic core F(1) (sensu Eukaryota)	**x**		**x**		**x**	**x**	4
	↓ Transcription factor TFIIH complex			**x**	**x**	**x**	**x**	4
	Cytoplasm	**x**	**x**			**x**	**x**	4
	↓ Proton-transporting ATP synthase, catalytic core (sensu Eukaryota)	**x**		**x**	**x**	**x**		4
	Cytosolic small ribosomal subunit (sensu Eukaryota)	**x**			**x**	**x**	**x**	4
	Cytoskeleton		**x**	**x**		**x**	**x**	4
	Isoamylase complex		**x**		**x**	**x**	**x**	4
	↓ Proton-transporting ATP synthase complex	**x**		**x**		**x**	**x**	4
	↓ Proton-transporting ATP synthase complex, catalytic core F(1)	**x**		**x**	**x**	**x**		4
	↓ Proton-transporting ATP synthase, catalytic core	**x**		**x**	**x**	**x**		4
	↓ Respiratory chain complex III	**x**	**x**			**x**	**x**	4

**Metabolic Pathways**	***Energy Metabolism***							
	↓ Reductive carboxylate cycle (CO2 fixation)	**x**	**x**	**x**	**x**	**x**	**x**	6
	↓ Oxidative phosphorylation	**x**	**x**	**x**		**x**		4
	↓ ATP synthesis	**x**		**x**		**x**		3
	↓ Carbon fixation		**x**			**x**		2
	***Carbohydrate Metabolism***							
	Glyoxylate and dicarboxylate metabolism	**x**	**x**	**x**		**x**		4
	↓ Inositol metabolism	**x**	**x**			**x**	**x**	4
	Glycolysis/Gluconeogenesis		**x**			**x**		2
	↓ Pentose phosphate pathway		**x**			**x**		2
	***Biosynthesis and secondary metabolism ***							
	Streptomycin biosynthesis		**x**		**x**	**x**		3
	Alkaloid biosynthesis I and II	**x**	**x**	**x**	**x**	**x**	**x**	6
	***Other***							
	Ribosome	**x**		**x**		**x**	**x**	4
	Apoptosis		**x**		**x**		**x**	3
	Chondroitin/Heparan sulfate biosynthesis	**x**		**x**	**x**			3
	D-Arginine and D-ornithine metabolism	**x**			**x**	**x**		3
	Prion disease	**x**		**x**	**x**			3
	Protein export			**x**		**x**	**x**	3

^$ ^W, B, K, DP, SA and SF stand respectively for Wachtel, Baer, Khan, De Pittà, Schaaf Adult and Schaaf Foetal datasets. Cells with an **x **represent enriched classes in the correspondent dataset.

^§ ^The presence of arrows (↑ up or ↓ down regulation) in the category name indicates the expression levels of genes belonging to the class.

**Table 4:** Entrez Gene entries and redundancy measure (RD) for each platform.

**Authors**	**Entrez Gene entries represented only once in the platform**		**RD**	**Total number of gene with Entrez Gene entry**		**Total number of gene without Entrez Gene entry**	
	*Actual number*	*Percentage*		*Actual number*	*Percentage*	*Actual number*	*Percentage*

Wachtel *et al*.	8,362	37.5	2.7	21,200	95.1	1,077	4.8
Baer *et al*.	7,378	58.4	2.4	11,998	95.0	627	5.0
De Pittà *et al*.	2,850	57.1	2.4	4,080	81.7	912	18.3
Khan *et al*.	2,029	88.1	2.0	2,035	88.4	268	11.6
Schaaf *et al*.	4,801	10.3	3.4	26,512	57.1	19,933	42.9

**Table 5:** Number of Entrez Gene entries shared by increasing number of expression studies (noted by *x*).

***x***	**# Entrez Genes entries shared by *x *studies**
1	4,876
2	4,617
3	4,087
4	2,125
5	594
