# Supplementary material for: Defining the gene expression signature of rhabdomyosarcoma by meta-analysis
Source: BMC Genomics. 2006 Nov 7;7:287. doi: 10.1186/1471-2164-7-287 (PMC1636648; doi:10.1186/1471-2164-7-287)
Supplement: Additional File 2 — Percentage and actual number of Entrez Genes entries shared by the studies included in the meta-analysis. Percentages are calculated according to the total number of probes per row. [file 1471-2164-7-287-S2.doc]

**Table S2*:*** **Percentage and actual number of Entrez Genes shared by studies used in the meta-analysis.**

| **Authors** | **Wachtel *et al.*** | **Baer *et al.*** | **De Pittà *et al.*** | **Khan *et al.*** | **Schaaf *et al.*** |
| --- | --- | --- | --- | --- | --- |
| **Wachtel *et al.*** | 100 | 35(7,803) | 12(2,682) | 8.7(1,944) | 38.5(8,599) |
| **Baer *et al.*** | 62.1(7,803) | 100 | 15.4(1,941) | 13.6(1,707) | 45(5,659) |
| **De Pittà *et al.*** | 53.7(2,682) | 38.9(1,941) | 100 | 15(745) | 54.5(2,721) |
| **Khan *et al.*** | 84(1,944) | 74(1,707) | 32.2(745) | 100 | 76.5(1,767) |
| **Schaaf *et al.*** | 18.5(8,599) | 12.1(5,659) | 6(2,721) | 4(1,767) | 100 |

Percentages are calculated according to the total number of probes *per* row. For instance:

7,803 Entrez Gene entries common between Wachtel and Baer dataset correspond to (i) 35% of the total number of probes in Wachtel’s study (7,803/ 22,283) and to (ii) 62.1% of the total number of probes in Baer’s study (7,803/ 12,558).
