# Supplementary material for: Defining the gene expression signature of rhabdomyosarcoma by meta-analysis
Source: BMC Genomics. 2006 Nov 7;7:287. doi: 10.1186/1471-2164-7-287 (PMC1636648; doi:10.1186/1471-2164-7-287)
Supplement: Additional File 3 — Complete list of differentially expressed meta-profiles. D1 and D2 columns with gray cells represents genes found differentially expressed in Davicioni et al. (2006) study. D1 column shows genes differentially expressed taking into account only embrional and alveolar RMS, while D2 shows gene differentially expressed with all the samples in Davicioni's paper. [file 1471-2164-7-287-S3.doc]

**Table S3**: *Complete list of differentially expressed meta-profiles. D1 and D2 columns with gray cells represents genes found differentially expressed in Davicioni et al. (2006) study. D1 column shows genes differentially expressed taking into account only embrional and alveolar RMS, while D2 shows gene differentially expressed with all the samples in Davicioni’s paper.*

| **Entrez Gene** | **Gene Description** | **D1** | **D2** | **Gene Symbol** | **Chr. Position** | **Comments** |  | | | |
| --- | --- | --- | --- | --- | --- | --- | --- | --- | --- | --- |
| **Underexpressed** | | | | | | |  | | | |
| 230 | **aldolase C, fructose-bisphosphate** |  |  | ALDOC | chr17cen-q12 |  |  | | | |
| 288 | **Ankyrin 3, node of Ranvier (ankyrin G)** |  |  | ANK3 | chr10q21 | **Muscle differentiation and sarcomeric morphogenesis** |  | | | |
| 2805 | **aspartate aminotransferase 1** |  |  | GOT1 | chr10q24.1-q25.1 |  |  | | | |
| 513 | **ATP synthase, H+ transporting, mitochondrial F1 complex, delta subunit** |  |  | ATP5D | chr19p13.3 | **Mitochondrion** |  | | | |
| 509 | **ATP synthase, H+ transporting, mitochondrial F1 complex, gamma polypeptide 1** |  |  | ATP5C1 | chr10p15.1 | **Mitochondrion** |  | | | |
| 9114 | **ATPase, H+ transporting, lysosomal, V0 subunit D** |  |  | ATP6V0D1 | chr16q22 | **Mitochondrion** |  | | | |
| 274 | **bridging integrator 1 isoform 8** |  |  | BIN1 | chr2q14 | **Already found deregulated in RMS** |  | | | |
| 10519 | **calcium and integrin binding 1 (calmyri n)** |  |  | CIB1 | chr15q25.3-q26 |  |  | | | |
| 782 | **calcium channel, voltage-dependent, beta 1 subunit** |  |  | CACNB1 | chr17q21-q22 |  |  | | | |
| 825 | **calpain 3 isoform a** |  |  | CAPN3 | chr15q15.1-q21.1 | **Muscle differentiation and sarcomeric morphogenesis** |  | | | |
| 844 | **calsequestrin 1 (fast-twitch, skeletal muscle)** |  |  | CASQ1 | chr1q21 | **Muscle differentiation and sarcomeric morphogenesis** |  | | | |
| 845 | **calsequestrin 2 (cardiac muscle)** |  |  | CASQ2 | chr1p13.3-p11 | **Muscle differentiation and sarcomeric morphogenesis** |  | | | |
| 5569 | **cAMP-dependent protein kinase inhibitor alpha** |  |  | PKIA | chr8q21.11 |  |  | | | |
| 10486 | **CAP, adenylate cyclase-associated protein, 2 (yeast)** |  |  | CAP2 | chr6p22.3 |  |  | | | |
| 761 | **carbonic anhydrase III, muscle specific** |  |  | CA3 | chr8q13-q22 |  |  | | | |
| 842 | **caspase 9, apoptosis-related cysteine protease** |  |  | CASP9 | chr1p36.3-p36.1 |  |  | | | |
| 1514 | **cathepsin L** |  |  | CTSL | chr9q21-q22 |  |  | | | |
| 8531 | **cold shock domain protein A** |  |  | CSDA | chr12p13.1 |  |  | | | |
| 10063 | **COX17 homolog, cytochrome c oxidase assembly** |  |  | COX17 | chr3q13.33 | **Mitochondrion** |  | | | |
| 8048 | **cysteine and glycine-rich protein 3** |  |  | CSRP3 | chr11p15.1 |  |  | | | |
| 1339 | **cytochrome c oxidase subunit VIa polypeptide 2** |  |  | COX6A2 | chr16p | **Mitochondrion** |  | | | |
| 1345 | **cytochrome c oxidase subunit VIc** |  |  | COX6C | chr8q22-q23 | **Mitochondrion** |  | | | |
| 1604 | **decay accelerating factor for complement (CD55, Cromer blood group system)** |  |  | DAF | chr1q32 |  |  | | | |
| 1603 | **defender against cell death 1** |  |  | DAD1 | chr14q11-q12 | **Anti-apoptosis, tumor progression and growth** |  | | | |
| 1725 | **deoxyhypusine synthase isoform a** |  |  | DHPS | chr19p13.2-p13.1 |  |  | | | |
| 1774 | **deoxyribonuclease I-like 1** |  |  | DNASE1L1 | chrXq28 |  |  | | | |
| 25874 | **DKFZP564B167 protein** |  |  | DKFZP564B167 | chr1q24 |  |  | | | |
| 3337 | **DnaJ (Hsp40) homolog, subfamily B, member 1** |  |  | DNAJB1 | chr19p13.2 |  |  | | | |
| 1632 | **dodecenoyl-Coenzyme A delta isomerase (3,2 trans-enoyl-Coenzyme A isomerase)** |  |  | DCI | chr16p13.3 |  |  | | | |
| 1778 | **dynein, cytoplasmic, heavy polypeptide 1** |  |  | DNCH1 | chr14q32.3-qter|14q32 |  |  | | | |
| 2050 | **ephrin receptor EphB4 precursor** |  |  | EPHB4 | chr7q22 |  |  | | | |
| 2039 | **erythrocyte membrane protein band 4.9 (dematin)** |  |  | EPB49 | chr8p21.1 |  |  | | | |
| 2101 | **estrogen-related receptor alpha** |  |  | ESRRA | chr11q13 |  |  | | | |
| 4632 | **fast skeletal myosin alkali light chain 1** |  |  | MYL1 | chr2q33-q34 | **Already found deregulated in RMS, Muscle differentiation and sarcomeric morphogenesis** |  | | | |
| 2170 | **fatty acid binding protein 3** |  |  | FABP3 | chr1p33-p32 |  |  | | | |
| 2354 | **FBJ murine osteosarcoma viral oncogene homolog** |  |  | FOSB | chr19q13.32 | **Anti-apoptosis, tumor progression and growth** |  | | | |
| 2273 | **four and a half LIM domains 1** |  |  | **FHL1** | chrXq26 | **Muscle differentiation and sarcomeric morphogenesis** |  | | | |
| 5348 | **FXYD domain containing ion transport regulator 1 (phospholemman)** |  |  | FXYD1 | chr19q13.1 | **Already found deregulated in RMS** |  | | | |
| 2870 | **G protein-coupled receptor kinase 6 isoform C** |  |  | GRK6 | chr5q35 |  |  | | | |
| 2058 | **glutamyl-prolyl-tRNA synthetase** |  |  | EPRS | chr1q41-q42 |  |  | | | |
| 2997 | **glycogen synthase 1 (muscle)** |  |  | GYS1 | chr19q13.3 |  |  | | | |
| 2593 | **guanidinoacetate N-methyltransferase** |  |  | GAMT | chr19p13.3 |  |  | | | |
| 2766 | **guanosine monophosphate reductase** |  |  | GMPR | chr6p23 |  |  | | | |
| 10456 | **HCLS1 associated protein X-1** |  |  | HAX1 | chr1q21.3 |  |  | | | |
| 27129 | **heat shock 27kDa protein family, member 7 (cardiovascular)** |  |  | HSPB7 | chr1p36.23-p34.3 |  |  | | | |
| 10524 | **HIV-1 Tat interacting protein, 60kDa** |  |  | HTATIP | chr11q13 |  |  | | | |
| 8692 | **hyaluronoglucosaminidase 2** |  |  | HYAL2 | chr3p21.3 |  |  | | | |
| 3295 | **hydroxysteroid (17-beta) dehydrogenase 4** |  |  | HSD17B4 | chr5q21 |  |  | | | |
| 3476 | **immunoglobulin binding protein 1** |  |  | IGBP1 | chrXq13.1-q13.3 |  |  | | | |
| 25802 | **leiomodin 1 (smooth muscle)** |  |  | LMOD1 | chr1q32 |  |  | | | |
| 10489 | **leucine rich repeat containing 41** |  |  | LRRC41 | chr1p34.1 |  |  | | | |
| 10233 | **leucine-rich B7 protein isoform 2** |  |  | B7 | chr12p13 |  |  | | | |
| 4633 | **myosin light chain 2** |  |  | MYL2 | chr12q23-q24.3 | **Muscle differentiation and sarcomeric morphogenesis** |  | | | |
| 4713 | **NADH dehydrogenase (ubiquinone) 1 beta** |  |  | NDUFB7 | chr19p13.12-p13.11 | **Mitochondrion** |  | | | |
| 4719 | **NADH dehydrogenase (ubiquinone) Fe-S protein 1,** |  |  | NDUFS1 | chr2q33-q34 | **Mitochondrion** |  | | | |
| 4724 | **NADH dehydrogenase (ubiquinone) Fe-S protein 4,** |  |  | NDUFS4 | chr5q11.1 | **Mitochondrion** |  | | | |
| 4779 | **nuclear factor (erythroid-derived 2)-like 1** |  |  | NFE2L1 | chr17q21.3 |  |  | | | |
| 4792 | **nuclear factor of kappa light polypeptide gene** |  |  | NFKBIA | chr14q13 | **Anti-apoptosis, tumor progression and growth** |  | | | |
| 8031 | **nuclear receptor coactivator 4** |  |  | NCOA4 | chr10q11.2 |  |  | | | |
| 2063 | **nuclear receptor subfamily 2, group F, member 6** |  |  | NR2F6 | chr19p13.1 |  |  | | | |
| 5264 | **phytanoyl-CoA hydroxylase (Refsum disease)** |  |  | PHYH | chr10pter-p11.2 |  |  | | | |
| 754 | **pituitary tumor-transforming 1 interacting protein** |  |  | PTTG1IP | chr21q22.3 |  |  | | | |
| 5687 | **proteasome alpha 6 subunit** |  |  | PSMA6 | chr14q13 |  |  | | | |
| 5164 | **pyruvate dehydrogenase kinase, isoenzyme 2** |  |  | PDK2 | chr17q21.33 | **Mitochondrion** |  | | | |
| 388 | **ras homolog gene family, member B** |  |  | RHOB | chr2p24 | **Anti-apoptosis, tumor progression and growth** |  | | | |
| 10670 | **Ras-related GTP binding A** |  |  | RRAGA | chr9p22.1 |  |  | | | |
| 5524 | **regulatory subunit PR 53 of protein phosphatase** |  |  | PPP2R4 | chr9q34 |  |  | | | |
| 55544 | **RNA-binding region containing protein 1 isoform** |  |  | RNPC1 | chr20q13.32 |  |  | | | |
| 10768 | **S-adenosylhomocysteine hydrolase-like 1** |  |  | AHCYL1 | chr1p13.2 |  |  | | | |
| 1160 | **sarcomeric mitochondrial creatine kinase** |  |  | CKMT2 | chr5q13.3 | **Muscle differentiation and sarcomeric morphogenesis** |  | | | |
| 6414 | **selenoprotein P, plasma, 1** |  |  | SEPP1 | chr5q31 |  |  | | | |
| 10430 | **seven transmembrane domain protein** |  |  | NIFIE14 | chr19q13.1 |  |  | | | |
| 89 | **skeletal muscle specific actinin, alpha 3** |  |  | ACTN3 | chr11q13-q14 |  |  | | | |
| 6575 | **solute carrier family 20, member 2** |  |  | **SLC20A2** | chr8p12-q21 | **Mitochondrion** |  | | | |
| 5250 | **solute carrier family 25 member 3 isoform b** |  |  | **SLC25A3** | chr12q23 | **Mitochondrion** |  | | | |
| 291 | **solute carrier family 25 mitochondrial carrier;** |  |  | **SLC25A4** | chr4q35 | **Mitochondrion** |  | | | |
| 6786 | **stromal interaction molecule 1 precursor** |  |  | **STIM1** | chr11p15.5 | **Anti-apoptosis, tumor progression and growth** |  | | | |
| 6649 | **superoxide dismutase 3, extracellular** |  |  | SOD3 | chr4p16.3-q21 |  |  | | | |
| 6840 | **Supervillin** |  |  | SVIL | chr10p11.2 |  |  | | | |
| 7123 | **tetranectin (plasminogen binding protein)** |  |  | TNA | chr3p22-p21.3 |  |  | | | |
| 9322 | **thyroid hormone receptor interactor 10** |  |  | TRIP10 | chr19p13.3 |  |  | | | |
| 7168 | **tropomyosin 1 (alpha)** |  |  | TPM1 | chr15q22.1 | **Muscle differentiation and sarcomeric morphogenesis** |  | | | |
| 7169 | **tropomyosin 2 (beta)** |  |  | TPM2 | chr9p13.2-p13.1 | **Muscle differentiation and sarcomeric morphogenesis** |  | | | |
| 7125 | **troponin C2, fast** |  |  | TNNC2 | chr20q12-q13.11 | **Already found deregulated in RMS, Muscle differentiation and sarcomeric morphogenesis** |  | | | |
| 7328 | **ubiquitin-conjugating enzyme E2H isoform 1** |  |  | UBE2H | chr7q32 |  |  | | | |
| 5887 | **UV excision repair protein RAD23 homolog B** |  |  | RAD23B | chr9q31.2 |  |  | | | |
| 2114 | **v-ets erythroblastosis virus E26 oncogene homolog 2 (avian)** |  |  | ETS2 | chr21q22.3|21q22.2 |  |  | | | |
| 596 | **B-cell CLL/lymphoma 2** |  |  | **BCL2** | chr18q21.33|18q21.3 |  |  | | | |
| **Overexpressed** | | | | | | | |  |  |  |
| 10109 | **actin related protein 2/3 complex, subunit 2, 34kDa** |  |  | ARPC2 | chr2q36.1 |  |  | | | |
| 10487 | **adenylyl cyclase-associated protein** |  |  | CAP1 | chr1p34.2 |  |  | | | |
| 23204 | **ADP-ribosylation factor-like 6 interacting** |  |  | ARL6IP | chr16p12-p11.2 | **Anti-apoptosis, tumor progression and growth** |  | | | |
| 10950 | **B-cell translocation gene 3** |  |  | BTG3 | chr21q21.1-q21.2 |  |  | | | |
| 1152 | **brain creatine kinase** |  |  | CKB | chr14q32 | **Mitochondrion** |  | | | |
| 811 | **calreticulin precursor** |  |  | CALR | chr19p13.3-p13.2 |  |  | | | |
| 977 | **CD151 antigen** |  |  | CD151 | chr11p15.5 | **Anti-apoptosis, tumor progression and growth** |  | | | |
| 10983 | **cyclin I** |  |  | CCNI | chr4q21.1 |  |  | | | |
| 1660 | **DEAH (Asp-Glu-Ala-His) box polypeptide 9** |  |  | DHX9 | chr1q25 |  |  | | | |
| 1839 | **diphtheria toxin receptor (heparin-binding** |  |  | DTR | chr5q23 |  |  | | | |
| 1650 | **dolichyl-diphosphooligosaccharide-protein** |  |  | DDOST | chr1p36.1 |  |  | | | |
| 11100 | **E1B-55kDa-associated protein 5 isoform a** |  |  | HNRPUL1 | chr19q13.2 |  |  | | | |
| 1997 | **E74-like factor 1 (ets domain transcription** |  |  | ELF1 | chr13q13 |  |  | | | |
| 3646 | **eukaryotic translation initiation factor 3, subunit 6 48kDa** |  |  | EIF3S6 | chr8q22-q23 |  |  | | | |
| 1973 | **Eukaryotic translation initiation factor 4A, isoform 1** |  |  | EIF4A1 | chr17p13 |  |  | | | |
| 2130 | **Ewing sarcoma breakpoint region 1 isoform EWS** |  |  | EWSR1 | chr22q12.2 | **Anti-apoptosis, tumor progression and growth** |  | | | |
| 2091 | **fibrillarin** |  |  | FBL | chr19q13.1 |  |  | | | |
| 2719 | **glypican 3** |  |  | GPC3 | chrXq26.1 |  |  | | | |
| 3020 | **H3 histone, family 3A** |  |  | H3F3A | chr1q41 |  |  | | | |
| 3146 | **high-mobility group box 1** |  |  | HMGB1 | chr13q12 |  |  | | | |
| 10919 | **HLA-B associated transcript 8 BAT8 isoform a** |  |  | BAT8 | chr6p21.31 |  |  | | | |
| 3275 | **HMT1 hnRNP methyltransferase-like 1** |  |  | HRMT1L1 | chr21q22.3 |  |  | | | |
| 10989 | **inner membrane protein, mitochondrial (mitofilin)** |  |  | IMMT | chr2p11.2|2 |  |  | | | |
| 3481 | **insulin-like growth factor 2 (somatomedin A)** |  |  | **IGF2** | chr11p15.5 | **Already found deregulated in RMS** |  | | | |
| 10945 | **KDEL (Lys-Asp-Glu-Leu) endoplasmic reticulum** |  |  | KDELR1 | chr19q13.3 |  |  | | | |
| 3921 | **laminin receptor 1** |  |  | LAMR1 | chr3p22.2 |  |  | | | |
| 9782 | **matrin 3** |  |  | MATR3 | chr5q31.2 |  |  | | | |
| 4281 | **midline 1 isoform alpha** |  |  | MID1 | chrXp22 |  |  | | | |
| 9242 | **musculin (activated B-cell factor-1)** |  |  | MSC | chr8q21 |  |  | | | |
| 4666 | **nascent-polypeptide-associated complex alpha** |  |  | NACA | chr12q23-q24.1 |  |  | | | |
| 4841 | **non-POU domain containing, octamer-binding** |  |  | NONO | chrXq13.1 |  |  | | | |
| 4833 | **nucleoside-diphosphate kinase 4** |  |  | NME4 | chr16p13.3 |  |  | | | |
| 5478 | **peptidylprolyl isomerase A (cyclophilin A)** |  |  | PPIA | chr7p13-p11.2 |  |  | | | |
| 9360 | **peptidyl-prolyl isomerase G (cyclophilin G)** |  |  | PPIG | chr2q31.1 |  |  | | | |
| 7001 | **peroxiredoxin 2 isoform a** |  |  | PRDX2 | chr19p13.2 |  |  | | | |
| 5226 | **phosphogluconate dehydrogenase** |  |  | PGD | chr1p36.3-p36.13 |  |  | | | |
| 8682 | **phosphoprotein enriched in astrocytes 15** |  |  | PEA15 | chr1q21.1 | **Anti-apoptosis, tumor progression and growth** |  | | | |
| 5764 | **pleiotrophin** |  |  | PTN | chr7q33-q34 |  |  | | | |
| 5216 | **profilin 1** |  |  | PFN1 | chr17p13.3 |  |  | | | |
| 5245 | **prohibitin** |  |  | PHB | chr17q21 |  |  | | | |
| 5695 | **proteasome (prosome, macropain) subunit, beta type, 7** |  |  | PSMB7 | chr9q34.11-q34.12 |  |  | | | |
| 5688 | **proteasome alpha 7 subunit isoform 1** |  |  | PSMA7 | chr20q13.33 |  |  | | | |
| 9647 | **protein phosphatase 1F (PP2C domain containing)** |  |  | PPM1F | chr22q11.22 |  |  | | | |
| 7879 | **RAB7, member RAS oncogene family** |  |  | RAB7 | chr3q21.3 |  |  | | | |
| 6157 | **ribosomal protein L27a** |  |  | RPL27A | chr11p15 |  |  | | | |
| 11224 | **ribosomal protein L35** |  |  | RPL35 | chr9q34.1 |  |  | | | |
| 6128 | **ribosomal protein L6** |  |  | RPL6 | chr12q24.1 |  |  | | | |
| 5936 | **RNA binding motif protein 4** |  |  | RBM4 | chr11q13 |  |  | | | |
| 6282 | **S100 calcium binding protein A11 (calgizzarin)** |  |  | S100A11 | chr1q21 |  |  | | | |
| 6627 | **small nuclear ribonucleoprotein polypeptide A'** |  |  | SNRPA1 | chr15q26.3 |  |  | | | |
| 6613 | **small ubiquitin-like modifier 2 isoform b** |  |  | SUMO2 | chr17q25.1 |  |  | | | |
| 292 | **solute carrier family 25, member 5** |  |  | **SLC25A5** | chrXq24-q26 | **Mitochondrion** |  | | | |
| 293 | **solute carrier family 25, member A6** |  |  | **SLC25A6** | chrXp22.32 and Yp | **Mitochondrion** |  | | | |
| 6653 | **sortilin-related receptor containing LDLR class** |  |  | SORL1 | chr11q23.2-q24.2 |  |  | | | |
| 6421 | **Splicing factor proline/glutamine rich (polypyrimidine tract binding protein associated)** |  |  | SFPQ | chr1p34.3 |  |  | | | |
| 6428 | **splicing factor, arginine/serine-rich 3** |  |  | SFRS3 | chr6p21 |  |  | | | |
| 8683 | **splicing factor, arginine/serine-rich 9** |  |  | SFRS9 | chr12q24.31 |  |  | | | |
| 27044 | **staphylococcal nuclease domain containing 1** |  |  | SND1 | chr7q31.3 |  |  | | | |
| 3925 | **stathmin 1** |  |  | STMN1 | chr1p36.1-p35 |  |  | | | |
| 10963 | **stress-induced-phosphoprotein 1** |  |  | STIP1 | chr11q13 |  |  | | | |
| 9168 | **thymosin, beta 10** |  |  | TMSB10 | chr2p11.2 |  |  | | | |
| 203068 | **tubulin, beta polypeptide** |  |  | TUBB | chr6p21.33 | **Muscle differentiation and sarcomeric morphogenesis** |  | | | |
| 7297 | **tyrosine kinase 2** |  |  | TYK2 | chr19p13.2 |  |  | | | |
| 9040 | **ubiquitin-conjugating enzyme E2M** |  |  | UBE2M | chr19q13.43 |  |  | | | |
| 7419 | **voltage-dependent anion channel 3** |  |  | VDAC3 | chr8p11.2 |  |  | | | |
